# Supplementary material for: Reconciling Mining with the Conservation of Cave Biodiversity: A Quantitative Baseline to Help Establish Conservation Priorities
Source: PLoS One. 2016 Dec 20;11(12):e0168348. doi: 10.1371/journal.pone.0168348 (PMC5173368; doi:10.1371/journal.pone.0168348)
Supplement: S1 Dataset — (ZIP) [file pone.0168348.s002.zip › Taxa/Serra Norte/SN_2013/PES/Lista PESE-04.pdf]

CAVIDADE PESE-0004

Classe	Ordem	Fam/Outros	Gên/Outros	Espécie	S-U	U-S	Zon.
Arachnida	Acari	Opilioacaridae	*Neoacarus*		X		E
Arachnida	Amblypygi	Phrynidae	*Heterophrynus*	*longicornis*	1	1	E/P
Arachnida	Araneae	Araneidae		jovem	X		E
Arachnida	Araneae	Ochyroceratidae	*Speocera*	sp.1	X		E
Arachnida	Araneae	Pholcidae	*Mesabolivar*	*aurantiacus*	X	X	E
Arachnida	Araneae	Prodidomidae	*Lygromma*	sp.2	X		E
Arachnida	Araneae	Theridiidae	*Theridion*	sp.1		X	P
Arachnida	Araneae	Theridiidae		sp.2	X	X	E/P
Arachnida	Araneae	Theridiosomatidae		sp.1	X		E
Arachnida	Opiliones	Escadabiidae		sp.4	X		E
Arachnida	Opiliones	Sclerosomatidae	*Prionostema*	sp.1	X	X	E/P
Diplopoda	Polydesmida	Fuhrmannodesmidae		sp.1	X	X	E/P
Entognatha	Collembola	Entomobryidae		sp.1	X		E
Entognatha	Collembola	Entomobryidae		sp.6		X	E
Entognatha	Collembola	Paronellidae		sp.1	X		E
Entognatha	Diplura	Campodeidae		sp.1	X		E
Insecta	Archaeognatha	Meinertellidae		sp.1		X	E
Insecta	Coleoptera	Carabidae		sp.3	X		E
Insecta	Coleoptera	Elateridae		jovem	X		E
Insecta	Coleoptera	Scydmaenidae		sp.1		X	E
Insecta	Coleoptera	Staphylinidae	Pselaphinae	sp.16	X	X	E
Insecta	Diptera	Cecidomyiidae	Cecidomyiinae	sp.		X	P
Insecta	Diptera	Chloropidae		sp.		X	E
Insecta	Diptera	Culicidae		jovem	X		E
Insecta	Diptera	Psychodidae	*Edentomyia*	*piauiensis*	X	X	E
Insecta	Diptera	Tipulidae	Tipulinae	sp.	X		E
Insecta	Heteroptera	Schizopteridae	Schizopterinae	jovem	X		E
Insecta	Homoptera	Fulgoromorpha		sp.1		X	E
Insecta	Hymenoptera	Chalcidoidea		sp.3		X	E
Insecta	Hymenoptera	Diapriidae		sp.1	X		E
Insecta	Hymenoptera	Formicidae	*Camponotus*	sp.1		X	E
Insecta	Hymenoptera	Formicidae	*Pachycondyla*	*striata*	X		E
Insecta	Hymenoptera	Formicidae	*Pheidole*	sp.2		X	E/P
Insecta	Hymenoptera	Formicidae	*Solenopsis*	sp.2	X	X	E
Insecta	Isoptera	Termitidae	*Nasutitermes*	sp.	X	X	E
Insecta	Lepidoptera	Noctuoidea		sp.4	X		E
Insecta	Orthoptera	Phalangopsidae	*Paraclodes*	sp.	2		E
Insecta	Psocoptera	Manicapsocidae	*Nothoentomum*	sp.1		X	E
Insecta	Psocoptera	Psocomorpha		jovem		X	P
Malacostraca	Isopoda	Platyarthridae		sp.1	X		E
Mammalia	Chiroptera	Emballonuridae	*Peropteryx*	sp.	1		E
Mammalia	Chiroptera	Phyllostomidae	*Carollia*	*perspicillata*		4	P
